# Comparison of lattice and pipeline flow diverters for the treatment of distal intracranial aneurysms: an inverse probability of treatment weighting analysis

**DOI:** 10.3389/fneur.2026.1851112

**Published:** 2026-07-09

**Authors:** Xinke Liu, Kaiyu Liu, Aoming Jin, Yan Zhao, Tian-Jie Lyu, Wenqi Hu, Yangyang Zhou, Qichen Peng, Xuanping Xie, Xiaoxi Zhu, Siyu Zhou, Jieran Huang, Xinjian Yang, Shiqing Mu

**Affiliations:** 1 Department of Interventional Neuroradiology, Beijing Neurosurgical Institute, Beijing Tiantan Hospital, Capital Medical University, Beijing, China; 2 China National Clinical Research Center for Neurological Diseases, Beijing Tiantan Hospital, Capital Medical University, Beijing, China; 3 School of Mechanical and Engineering, University of Science and Technology Beijing, Beijing, China; 4 Beijing Key Laboratory of Drug and Device Research and Development for Cerebrovascular Diseases, Beijing Tiantan Hospital, Capital Medical University, Beijing, China; 5 Department of Mechanical and Aerospace Engineering, The Hong Kong University of Science and Technology, Tai Po Tsai, Hong Kong SAR, China

**Keywords:** aneurysm, endovascular treatment, flow diverter, occlusion rates, the inflow angle of the aneurysm (IFAA)

## Abstract

**Background:**

Distal intracranial aneurysms are relatively rare in clinical practice and are characterized by distal location, smaller parent vessels, and fragile surrounding brain tissue, which are associated with a relatively higher risk of treatment-related complications such as ischemia and rebleeding. Traditional flow diverter stents have a thin tip, delivery wire can cause intraparenchymal hemorrhage or distal vessel perforation, particularly when attempting to navigate through complex, narrow, or fragile arteries. Therefore, the use of flow diverter devices in the treatment of distal intracranial aneurysms remains technically challenging. Flow diverter devices have been widely used for the treatment of wide-neck intracranial aneurysms; however, comparative data between the domestically developed Lattice device (Accu Medical, China) and the Pipeline Embolization Device remain limited. In this study, we compared the efficacy and safety of these two flow diverters in the treatment of unruptured distal intracranial aneurysms, based on scheduled postoperative imaging follow-up using computed tomography angiography (CTA) and digital subtraction angiography (DSA), focusing on aneurysm occlusion, complications, and branch vessel occlusion.

**Methods:**

This retrospective study included 47 patients with distal intracranial aneurysms treated with flow diverter devices between January 2020 and July 2025, who underwent implantation of either the Lattice or Pipeline stent. All patients underwent imaging follow-up at 6 months postoperatively using either computed tomography angiography (CTA) or digital subtraction angiography (DSA). Inverse probability weighting (IPTW) was performed to balance baseline characteristics between the two groups. Baseline characteristics before and after IPTW are presented in Table 1. After adjusting for age, sex, hypertension, diabetes, preoperative mRS, aneurysm location, aneurysm morphology (saccular vs. fusiform), neck type (wide vs. narrow), maximum aneurysm diameter, neck size, dome-to-neck ratio, the inflow angle of the aneurysm (IFAA), branch vessel diameter, branch vessel origin, and mean parent vessel diameter, propensity scores were calculated for inverse probability of treatment weighting (IPTW). We applied 1/PS as a weight for the Lattice group and 1/(1 − PS) for the Pipeline group. Inverse probability weighting was applied, and logistic regression analysis was performed to identify factors potentially associated with aneurysm occlusion. Aneurysm healing was assessed using the OKM grading scale, with grades 0–2 defined as poor occlusion and grades 3–4 defined as favorable occlusion.

**Results:**

Compared with the Pipeline group, no statistically significant differences were observed in aneurysm occlusion rates or branch vessel occlusion rates in the Lattice group at a median follow-up duration of 179 days (77.8% vs. 75.9%, *p* = 0.586). Among 18 patients treated with the Lattice device, 14 achieved favorable occlusion on follow-up imaging (14/18, 77.8%), whereas 22 of 29 patients treated with the Pipeline device demonstrated favorable occlusion (22/29, 75.9%). Branch vessel occlusion covered by the stent occurred in 3 patients in the Lattice group (3/18, 16.7%), which was slightly higher than that in the Pipeline group (2/29, 6.9%); however, this difference was not statistically significant (*p* = 0.317). Logistic regression analysis identified the IFAA as a potential factor associated with delayed or incomplete aneurysm healing. Specifically, for each 1-degree increase in this angle, the odds of aneurysm occlusion decreased by 3.5% (OR = 0.965, 95% CI: 0.933–0.998, *p* = 0.037). In addition, IPTW-adjusted univariate weighted logistic regression showed that larger aneurysm neck diameter (OR 0.685, 95% CI 0.505–0.929, *p* = 0.016), presence of branch vessel origin from aneurysms (OR 0.065, 95% CI 0.011–0.364, *p* = 0.003) were also associated with unfavorable OKM outcomes.

**Conclusion:**

After IPTW adjustment, Lattice and Pipeline devices demonstrated comparable efficacy and safety in the treatment of distal cerebral aneurysms. Aneurysm geometric and anatomical factors, including the inflow angle (IFAA), aneurysm neck diameter and branch vessel origin, were independently associated with incomplete or delayed aneurysm occlusion.

## Introduction

Distal cerebral artery aneurysms (DCA), in particular, aneurysms arising from the A2 (anterior cerebral artery), M2 (middle cerebral artery), and P2 (posterior cerebral artery) segments or more distal branches represent a small but high risk portion of intracranial aneurysms (1). Owing to the small vessel caliber, increased vascular tortuosity and broad base neck or fusiform like shape make it difficult for clipping or coiling (2–6).

**Figure 1:**
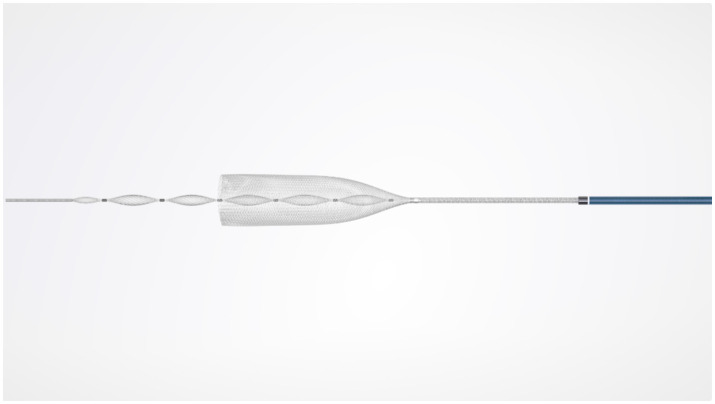
Lattice flow diverter (AccuMedical, Beijing).

Flow diverters (FD) which was designed to treat proximal internal carotid artery aneurysms has broaden the treatment of distal artery aneurysms. The broad base neck or fusiform shape make DCA more suitable for FD treatment. However, FD treatment DCA of remains controversial because the high perforation risk during FD deployment in such small caliber artery and the risk of infarction caused by branch artery occlusion. The Lattice Flow Diverter (Figure 1), created by AccuMedical in Beijing, is a novel device featuring two key innovations: a balloon-assisted delivery system that keeps the tip wire stable throughout the deployment, and a proprietary surface modification (MIROR) designed to reduce thrombogenic risk and promote endothelialization for improved vascular healing (7, 8).

Over the past 5 years, Pipeline flow-diverting stents have been explored in the treatment of distal intracranial aneurysms. The Pipeline and Lattice devices provide similar material and surface coverage (9–11). However, data regarding the safety and efficacy of lattice flow diverter in the management of distal intracranial aneurysms remain unexplored. This study aimed to evaluate and compare the safety and efficacy of two currently used flow diverters, Pipeline Flex and Lattice, in the treatment of distal artery aneurysms.

## Methods

### Patient selection

This retrospective study included 47 consecutive patients diagnosed with distal intracranial aneurysms at our center between January 2020 and July 2025 who were treated with either the Lattice or Pipeline device. The inclusion criteria were as follows: (1) age between 18 and 80 years; and (2) Diagnosis of a distal intracranial aneurysm located in either the anterior or posterior circulation and treated with the Lattice or Pipeline device (defined as aneurysms located beyond the A2, M2, or P2 segments, excluding those involving the M1 segment and bifurcation aneurysms). The exclusion criteria were: (1) ruptured aneurysms; (2) intracranial aneurysms previously treated with endovascular or neurosurgical intervention; (3) concomitant cerebrovascular diseases, including arteriovenous malformations, arteriovenous fistulas, or moyamoya disease; (4) poor imaging quality or absence of angiographic follow-up data.

### Procedure

Patients undergoing endovascular treatment received dual antiplatelet therapy for at least 5 days prior to the procedure, consisting of clopidogrel (75 mg/day) and aspirin (100 mg/day), along with platelet function testing. Patients with a platelet inhibition rate of less than 36.4% were considered clopidogrel-resistant and were switched to ticagrelor (12, 13). All procedures were performed under general anesthesia. The target aneurysm was accessed via a standard transfemoral approach. Postoperatively, clopidogrel (75 mg/day) was discontinued at 3–6 months, whereas aspirin (100 mg/day) was continued for 1 year.

### Three-dimensional reconstruction

Based on the original 3D-DSA data, vascular segmentation and three-dimensional reconstruction were performed using 3D Slicer (version 5.6.2, https://www.slicer.org/). The reconstructed surface models were subsequently smoothed, and irrelevant branches were removed. The final 3D models were independently evaluated by two senior interventional neuroradiologists with more than 5 years of experience to confirm successful reconstruction. Cases with discrepant assessments between the two neuroradiologists were excluded from the study.

### Centerline extraction and angular parameter calculation

**Figure 2:**
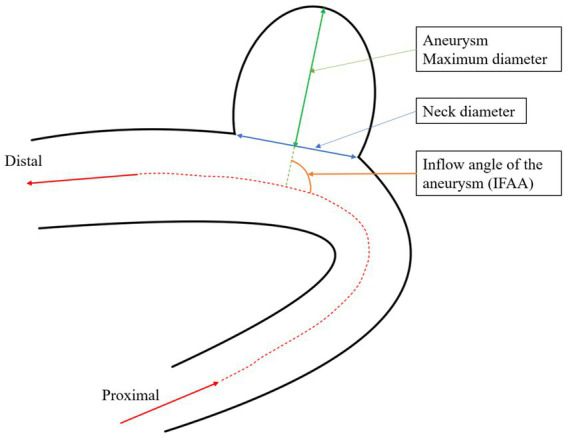
Schematic illustration of aneurysm morphology, including neck diameter, maximum aneurysm diameter, and inflow angle (IFAA).

**Figure 3:**
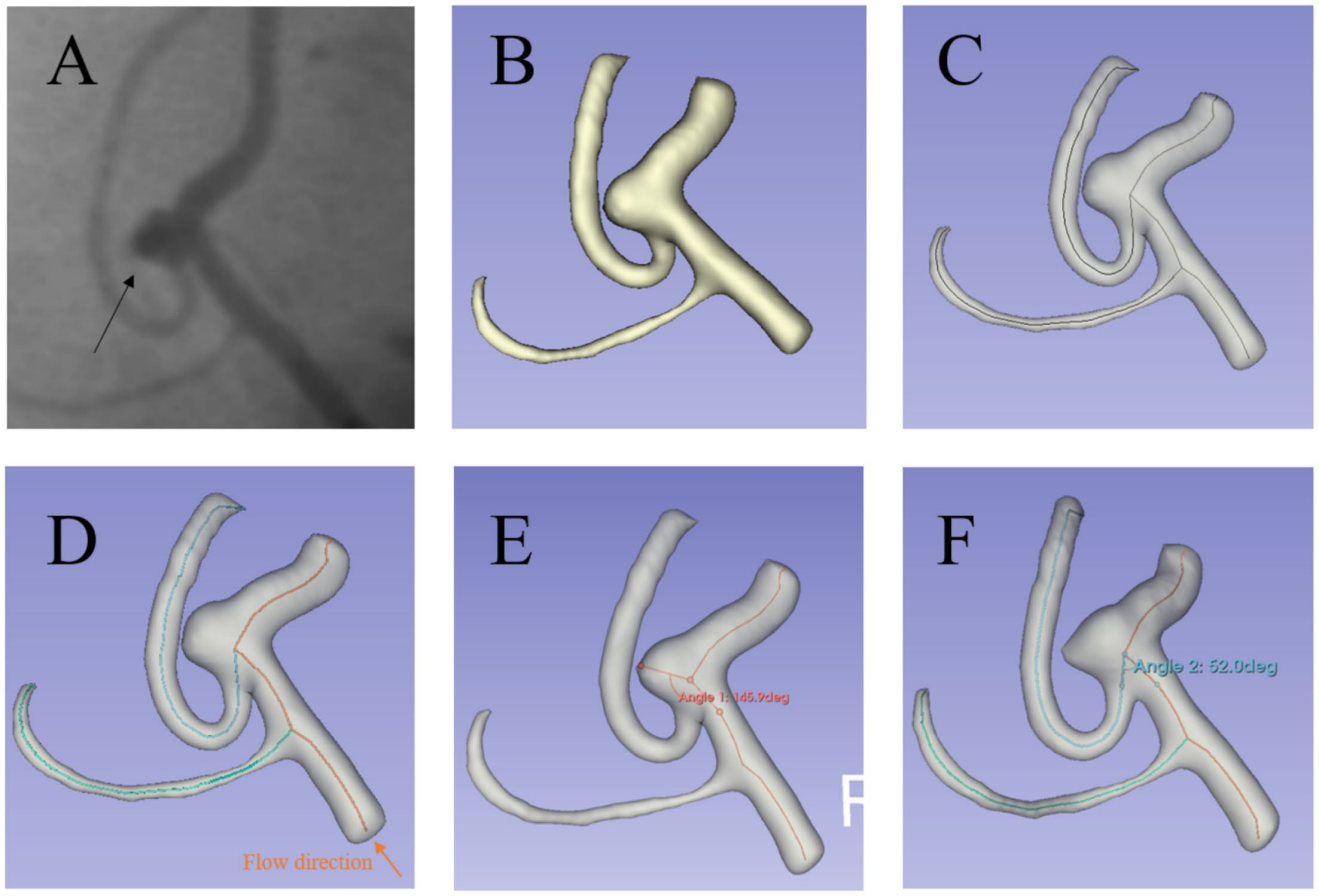
**(A)** Preoperative DSA image of an aneurysm located in the right anterior cerebral artery (arrow). **(B)** Three-dimensional reconstructed model of the vessel and aneurysm generated from the original DICOM data using segmentation and surface reconstruction tools. **(C)** Extraction of the arterial centerline using the VMTK extension. **(D)** The red line represents the parent vessel centerline, while the blue lines indicate the two branch vessels. **(E)** Measurement of the IFAA using the angle tool (Angle 1 = 145.9°). **(F)** Measurement of the IFAB (Angle 2 = 52.0°).

The centerline was extracted using the Vascular Modeling Toolkit (VMTK) extension implemented in 3D Slicer. The centerline was defined as the locus of the centers of the maximal-inscribed spheres along the vessel itself (14). Subsequently, the maximum aneurysm diameter, aneurysm neck diameter, dome-to-neck ratio, mean parent vessel diameter, and branch vessel diameter were calculated. Additionally, the angle between the parent vessel centerline and the maximum aneurysm diameter was defined as the inflow angle of the aneurysm (IFAA), whereas the angle between the parent vessel centerline and the branch vessel centerline was defined as the inflow angle of the branch artery (IFAB), as illustrated in Figures 2, 3.

### Hospitalization and outcomes

Demographic data included age, sex, body weight, height, and comorbidities (hypertension and diabetes mellitus). Aneurysm characteristics comprised aneurysm type, neck morphology, maximum aneurysm diameter, aneurysm neck diameter, neck size, dome-to-neck ratio, mean parent artery diameter, aneurysm location, branch vessel diameter, branch vessel origin the angle between the maximum aneurysm diameter and the centerline of the parent artery (IFAA), and the angle between the centerline of the branch vessel and that of the parent artery (IFAB). Branch vessel origin (BVO) was defined as the presence of a branch vessel arising from the aneurysm sac or originating from the parent artery segment covered by the implanted flow diverter. The anatomical relationship between branch vessels and the aneurysm was assessed using preoperative DSA and three-dimensional angiographic reconstructions. Procedural details, including device type and stent size, were also recorded. Angiographic and clinical outcomes during hospitalization and follow-up were systematically collected. Clinical and angiographic follow-up evaluations were scheduled at 6 months after treatment. In patients with complete aneurysm occlusion confirmed by digital subtraction angiography (DSA), annual imaging surveillance was performed using computed tomography angiography (CTA).

The primary follow-up outcome was aneurysm occlusion rate, which was assessed using the O’Kelly–Marotta (OKM) grading scale. Aneurysm occlusion was evaluated using the OKM grading scale. Secondary follow-up outcomes included the patency of jailed perforating or branch vessels after flow diverter deployment, the occurrence of in-stent stenosis, and clinical outcomes assessed by the modified Rankin Scale (mRS). Procedure-related complications, including ischemic and hemorrhagic events, were also recorded. Clinical status was evaluated according to the mRS score at follow-up, with scores ranging from 0 (no symptoms) to 6 (death).

### Statistical analysis

Statistical analysis was performed using R version 4.2.3 software, employing various packages such as gtsummary, matchIt, dplyr and readxl. Continuous variables were summarized using mean and standard deviation to describe baseline characteristics, while categorical variables were presented as counts and percentages. Continuous variables with a normal distribution were compared using the unpaired Student’s *t*-test, whereas non-normally distributed variables were analyzed using the Wilcoxon rank-sum test. Categorical variables were presented as counts and percentages and compared using the *χ*^2^ test or Fisher’s exact test, as appropriate.

We used Inverse probability weighting (IPTW) to address potential confounding factors and selection bias. Logistic regression analysis was performed to identify factors potentially associated with aneurysm occlusion. Postoperative aneurysm healing was assessed using the O’Kelly–Marotta (OKM) grading scale. OKM grades A, B and C were defined as incomplete occlusion, whereas OKM grades D and E were considered favorable occlusion. A two-sided *p*-value < 0.05 was considered statistically significant.

## Results

### Baseline data

A total of 47 patients with a mean age of 59 years were screened for the study. Of the participants, 55% were male and 45% were female. Among these patients, 18 were treated with the Lattice stent and 29 with the Pipeline device. Baseline characteristics were generally comparable between the two groups, except for differences in preoperative modified Rankin Scale (mRS) scores. Detailed baseline data of the two groups are presented in Table 1.

**Table 1:** Baseline characteristics of unmatched cohort.

Characteristic	Overall, *N* = 47^1^	Lattice, *N* = 18 (38%)^1^	Pipeline, *N* = 29 (62%)^1^	*p*-value^2^	SMD
Age	59.11 (11.06)	62.56 (10.92)	56.97 (10.78)	0.092	0.515
Sex				0.379	0.359
Female	21 (44.7%)	10 (55.6%)	11 (37.9%)		
Male	26 (55.3%)	8 (44.4%)	18 (62.1%)		
Weight	69.31 (13.67)	71.75 (14.91)	67.79 (12.89)	0.340	0.284
Height	166.47 (8.89)	165.89 (9.39)	166.83 (8.72)	0.729	0.104
Hypertension				0.482	0.308
No	20 (42.6%)	6 (33.3%)	14 (48.3%)		
Yes	27 (57.4%)	12 (66.7%)	15 (51.7%)		
Diabetes				0.470	0.347
No	38 (80.9%)	16 (88.9%)	22 (75.9%)		
Yes	9 (19.1%)	2 (11.1%)	7 (24.1%)		
Preoperative MRS				0.009	0.901
0	40 (85.1%)	12 (66.7%)	28 (96.6%)		
1	5 (10.6%)	5 (27.8%)	0 (0.0%)		
3	2 (4.3%)	1 (5.6%)	1 (3.4%)		
Aneurysm type				0.808	0.343
Fusiform	1 (2.1%)	1 (5.6%)	0 (0%)		
Saccular	46 (97.9%)	17 (94.4%)	29 (100%)		
Neck type				>0.9	0.025
Wide	42 (89.4%)	16 (88.9%)	26 (89.7%)		
Narrow	5 (10.6%)	2 (11.1%)	3 (10.3%)		
Max diameter	9.61 (22.13)	10.43 (23.38)	9.11 (21.72)	0.845	0.058
Neck diameter	4.46(2.07)	4.41(1.77)	4.48(2.26)	0.908	0.036
DNR (Dome-to-neck ratio)	2.87(8.97)	2.16(4.02)	3.32(11.04)	0.671	0.140
Parent artery mean diameter	1.93(0.46)	1.84(0.46)	1.98(0.46)	0.315	0.305
IFAA	107.28(30.94)	111.61(32.20)	104.59(30.39)	0.455	0.224
IFAB	83.83 (40.50)	78.39 (46.77)	87.21 (36.55)	0.474	0.210
Branch vessel origin				0.190	0.496
From aneurysms	12(25.5%)	7(38.9%)	5(17.2%)		
From stent-covered vascular segments	35(74.5%)	11(61.1%)	24(82.8%)		
Follow time	179 (161–245)	200 (163–347)	173 (160–207)	0.215	0.367
Branch diameter	1.20(0.45)	1.01(0.48)	1.31(0.39)	0.023	0.691
Location				0.702	0.469
A2	12 (25.5%)	6 (33.3%)	6 (20.7%)		
A3	11 (23.4%)	5 (27.8%)	6 (20.7%)		
A4	1 (2.1%)	0 (0%)	1 (3.4%)		
M2	17 (36.2%)	5 (27.8%)	12 (44.4%)		
P2	6 (12.8%)	2 (11.1%)	4 (13.8%)		
Stent diameter	2.55 (0.18)	2.55 (0.28)	2.54 (0.10)	0.902	0.033

^1^Continuous variables are presented as mean (SD) or median (IQR), and categorical variables as *n* (%).

^2^Wilcoxon rank sum test; Pearson’s Chi-squared test; Fisher’s exact test; Wilcoxon rank sum exact test.

**Figure 4:**
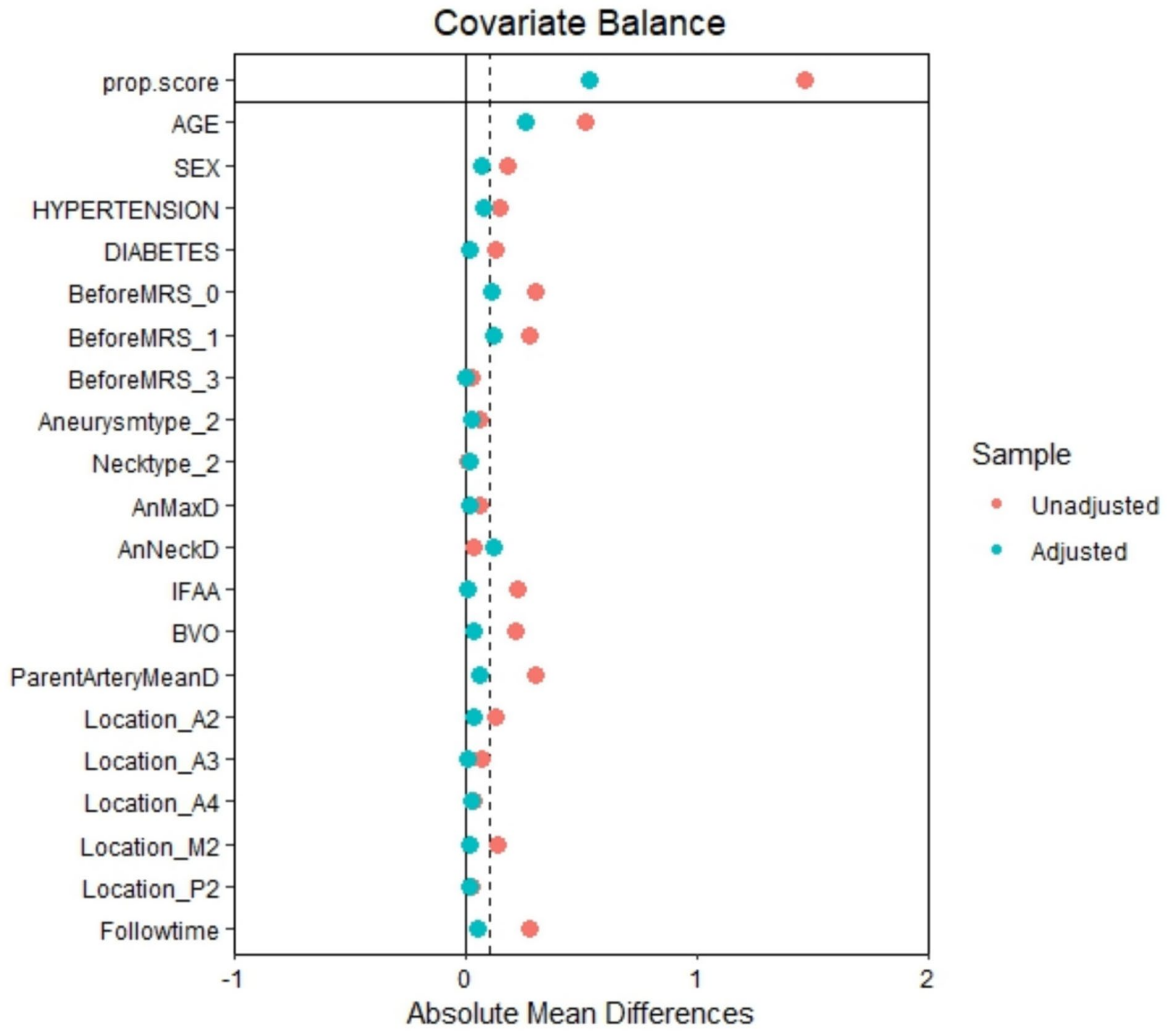
Standardized mean differences of variables before and after IPTW. BeforeMRS, Preoperative modified Rankin Scale; AnMaxD, aneurysm max diameter; AnNeckD, aneurysm neck diameter; IFAA, the inflow angle of the aneurysm; BVO, Branch vessel origin.

Stabilized inverse probability of treatment weighting (IPTW) based on propensity scores was applied to reduce baseline imbalance between groups. To minimize the influence of extreme weights and improve model stability, weight truncation was performed at the 1st and 99th percentiles. After adjusting for age, sex, hypertension, diabetes, preoperative MRS, aneurysm location, aneurysm morphology (saccular vs. fusiform), neck type (wide vs. narrow), maximum aneurysm diameter, neck size, IFAA, branch vessel diameter, branch vessel origin, follow time and mean parent vessel diameter, covariate balance was markedly improved, with most variables achieving SMDs below 0.1 (Figure 4). After IPTW, baseline characteristics were well balanced between the two groups, as shown in Table 2. Most standardized mean differences were below 0.1, indicating adequate covariate balance.

**Table 2:** Baseline characteristics after IPTW.

Characteristic	Overall, *N* = 41.29^1^	Lattice, *N* = 15.74 (38%)^1^	Pipeline, *N* = 25.56 (62%)^1^	*p*-value^2^	SMD
Age	58.58 (10.84)	59.81 (11.46)	57.82 (10.57)	0.595	0.181
Sex				0.556	0.101
Female	18.79 (45.52%)	8.15 (51.78%)	10.64 (41.65%)		
Male	22.50 (54.48%)	7.59 (48.22%)	14.91 (58.35%)		
Weight	69.93 (15.02)	75.33 (17.07)	66.60 (12.81)	0.124	0.578
Height	166.70 (9.23)	167.25 (10.01)	166.35 (8.88)	0.788	0.095
Hypertension				0.848	0.033
No	19.64 (47.55%)	7.16 (45.49%)	12.48 (48.83%)		
Yes	21.66 (52.45%)	8.58 (54.51%)	13.08 (51.17%)		
Diabetes				0.745	0.046
No	33.26 (80.55%)	13.13 (83.41%)	20.13 (78.78%)		
Yes	8.03 (19.45%)	2.61 (16.59%)	5.42 (21.22%)		
Preoperative MRS				–	–
0	38.12 (92.32%)	13.35 (84.78%)	24.78 (96.97%)		
1	1.91 (4.64%)	1.91 (12.16%)	0.00 (0.00%)		
3	1.26 (3.04%)	0.48 (3.06%)	0.78 (3.03%)		
Aneurysm type				0.232	0.024
Fusiform	0.38 (0.93%)	0.38 (2.43%)	0.00 (0.00%)		
Saccular	40.91 (99.07%)	15.36 (97.57%)	25.55 (100.00%)		
Neck type				0.945	0.008
Wide	36.44 (88.24%)	13.81 (87.74%)	22.62 (88.55%)		
Narrow	4.86 (11.76%)	1.93 (12.26%)	2.93 (11.45%)		
Max diameter	12.89 (29.06)	14.08 (30.33)	12.15 (28.76)	0.869	0.065
Neck diameter	4.24 (2.05)	4.10 (1.89)	4.33 (2.17)	0.734	0.110
DNR (Dome-to-neck ratio)	4.13 (11.90)	2.82 (5.14)	4.94 (14.64)	0.591	0.193
Parent artery mean diameter	1.94 (0.47)	1.85 (0.53)	2.00 (0.43)	0.390	0.310
IFAA	105.02 (33.26)	104.28 (31.73)	105.48 (34.71)	0.917	0.036
IFAB	83.81 (38.22)	76.62 (44.60)	88.25 (33.79)	0.368	0.294
Follow time	176 (162–240)	182 (110–346)	176 (162–226)	0.841	0.064
Branch diameter	1.23 (0.43)	1.07 (0.47)	1.33 (0.38)	0.064	0.591
Branch vessel origin				0.821	0.030
From aneurysms	8.80 (21.31%)	3.64 (23.15%)	5.15 (20.17%)		
From stent-covered vascular segments	32.50 (78.69%)	12.10 (76.85%)	20.40 (79.83%)		
Location				–	–
A2	10.43 (25.26%)	4.25 (26.98%)	6.18 (24.19%)		
A3	7.11 (17.21%)	2.67 (16.98%)	4.43 (17.36%)		
A4	0.62 (1.49%)	0.00 (0.00%)	0.62 (2.41%)		
M2	17.84 (43.19%)	6.66 (42.30%)	11.18 (43.74%)		
P2	5.30 (12.85%)	2.16 (13.74%)	3.14 (12.29%)		
Stent diameter	2.54 (0.19)	2.54 (0.28)	2.54 (0.10)	0.990	0.004

^1^Continuous variables are presented as mean (SD) or median (IQR), and categorical variables as *n* (%); Stabilized inverse probability weights were applied; therefore, weighted counts shown in the IPTW-adjusted cohort do not necessarily equal the original sample sizes.

^2^Design-based Kruskal–Wallis test.

Before IPTW, no statistically significant differences were observed between the Lattice and Pipeline groups with respect to aneurysm occlusion rates or branch vessel occlusion rates at a median follow-up duration of 179 days. In the Lattice-treated cohort, 14 of 18 patients achieved satisfactory aneurysm healing on scheduled imaging follow-up (14/18, 77.8%), whereas 22 of 29 patients treated with the Pipeline device demonstrated complete or satisfactory occlusion (22/29, 75.9%). There was no significant difference in satisfactory aneurysm occlusion rates between the two groups (77.8% vs. 75.9%, *p* = 0.586). Branch vessel occlusion covered by the stent was identified in 3 patients in the Lattice group (3/18, 16.7%), which was slightly higher than that observed in the Pipeline group (2/29, 6.9%); however, the difference did not reach statistical significance (*p* = 0.317). Among the five patients who developed branch vessel occlusion, 80% (4/5) had an acute branch angle (IFAB < 90°), suggesting a potential association between sharper branch angles and an increased risk of branch occlusion.

Regarding postoperative complications, one hemorrhagic event was observed in the Lattice group at 1-year follow-up (1/18, 5.6%), while two hemorrhagic complications occurred in the Pipeline group (2/29, 6.9%). No statistically significant difference in postoperative complications was found between the two groups.

**Figure 5:**
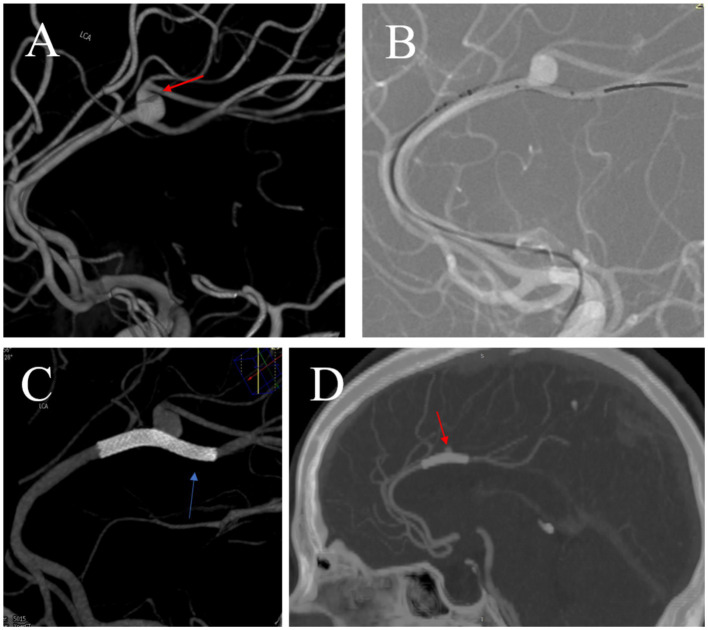
**(A)** A 56-year-old female patient with an aneurysm located in the A3 segment of the left anterior cerebral artery (red arrow). **(B)** Deployment of the Lattice stent under roadmap guidance. **(C)** Three-dimensional reconstructed image after deployment of a Lattice stent (blue arrow). **(D)** At 6-month follow-up, CTA showed that the aneurysm was not completely occluded (red arrow), and the two branch vessels originating from the aneurysm were occluded.

**Figure 6:**
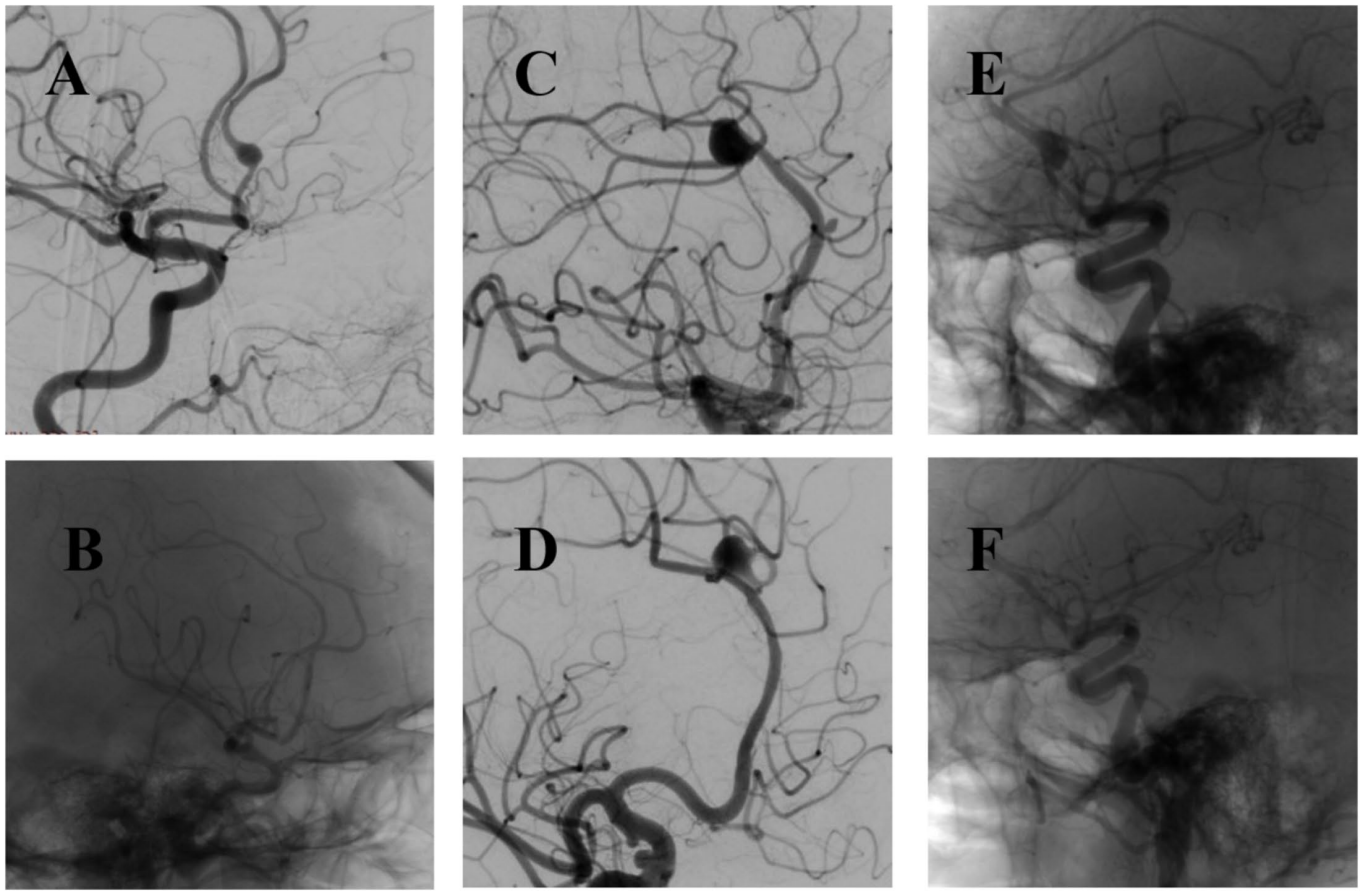
**(A)** Aneurysm located in the A2 segment of the anterior cerebral artery. **(B)** Digital subtraction angiography (DSA) at 6 months after treatment with the Lattice stent demonstrated complete aneurysm occlusion. **(C)** Aneurysm located in the A3 segment of the anterior cerebral artery. **(D)** DSA at 6 months after treatment with the Lattice stent indicated incomplete aneurysm occlusion. **(E)** Aneurysm located in the A2 segment of the anterior cerebral artery. **(F)** Six-month follow-up DSA after treatment with the Pipeline device demonstrated complete aneurysm occlusion.

After IPTW, there was no significant difference in the occlusion rate between the two groups (OR = 0.3, 95% CI: 0.072–1.546, *p* = 0.156). Logistic regression analysis indicated that the IFAA may be associated with delayed or incomplete aneurysm occlusion. Specifically, for each 1-degree increase in this angle, the odds of aneurysm occlusion decreased by 3.5% (OR = 0.965, 95% CI: 0.933–0.998, *p* = 0.037). In addition, we observed that IPTW-adjusted univariate weighted logistic regression demonstrated that larger aneurysm neck diameter (OR 0.685, 95% CI 0.505–0.929, *p* = 0.016), presence of branch vessel origin from aneurysms (OR 0.065, 95% CI 0.011–0.364, *p* = 0.003) were associated with unfavorable OKM outcomes (Table 3 and Figures 5, 6).

**Table 3:** IPTW-adjusted univariate weighted logistic regression.

Variable	OR (95% CI)	*P*-value
Stent: pipeline	0.333 (0.072–1.546)	0.156
Age	0.949 (0.89–1.012)	0.109
Sex: male	1.038 (0.216–5)	0.962
Hypertension	0.549 (0.093–3.263)	0.502
Diabetes	1.163 (0.178–7.573)	0.872
Max diameter	1.077 (0.905–1.282)	0.396
Neck diameter	0.685 (0.505–0.929)	0.016
DNR	9.353 (1.038–84.289)	0.046
Branch vessel origin: From aneurysms	0.065 (0.011–0.364)	0.003
IFAA	0.965 (0.933–0.998)	0.037
Branch diameter	0.77 (0.195–3.037)	0.703

IPTW-adjusted stratified sensitivity analyses demonstrated no significant interaction between stent type and IFAA, branch vessel configuration, hypertension, or diabetes mellitus (all *p* values for interaction > 0.05). The direction and magnitude of the effect of stent type on aneurysm occlusion were generally consistent across subgroups (Table 4).

**Table 4:** The impact of stent type on aneurysm healing among different subgroups.

Subgroup	OR	95% CI lower	95% CI upper	*P*-value	OR (95% CI)
IFAA ≤ 90°	1.096	0.360	3.338	0.874	1.096 (0.36–3.338)
IFAA > 90°	1.011	0.441	2.318	0.979	1.011 (0.441–2.318)
Branch vessel origin from aneurysms	0.751	0.210	2.690	0.669	0.751 (0.21–2.69)
Branch vessel origin from stent-covered vascular segments	1.152	0.529	2.507	0.724	1.152 (0.529–2.507)
Hypertension yes	0.952	0.418	2.166	0.908	0.952 (0.418–2.166)
Hypertension no	1.178	0.400	3.464	0.770	1.178 (0.4–3.464)
Diabetes yes	1.196	0.243	5.886	0.832	1.196 (0.243–5.886)
Diabetes no	1.010	0.484	2.107	0.979	1.01 (0.484–2.107)

## Discussion

The broad-necked or fusiform morphology of distal cerebral aneurysms (DCA) makes conventional treatment strategies, such as clipping or coiling, technically challenging. The use of flow diverters (FDs) in DCA remains controversial due to the risks associated with device deployment, including delivery wire–related perforation and ischemic complications from jailed branch vessels. Cimflova et al. (15) reported that flow diversion for distal aneurysms located in the M2 segment and beyond achieved complete or near-complete occlusion in 70% of patients at long-term follow-up. Previous meta-analyses have shown that, with a mean follow-up time of 12 months, flow diverter therapy for distal aneurysms achieves a complete/near-complete occlusion rate of approximately 70–90%, with procedure-related complications occurring in approximately 9% (16–18). The Lattice flow diverter, featuring a mechanically controlled balloon-assisted delivery system and MIROR surface modification, may be particularly suited for the treatment of distal intracranial aneurysms (DCA). Meanwhile, the Pipeline Flex and Lattice flow diverters share similar materials and comparable surface coverage. To date, no study has specifically evaluated the safety and efficacy of the Lattice flow diverter in DCA.

In this study, we compared the therapeutic outcomes of the Lattice flow diverter (AccuMedical, China) and the Pipeline Embolization Device (PED; Medtronic Inc., United States) in 47 patients with distal intracranial aneurysms. There were no significant differences between the Lattice flow diverter and the Pipeline stent in terms of 6-month aneurysm occlusion rates (77.8% vs. 75.9% *p* > 0.05), branch vessel occlusion, or procedure-related complications in the treatment of aneurysms beyond the Circle of Willis. Additionally, IFAA may be associated with aneurysm healing, whereas a larger vascular angle appeared to correlate with delayed aneurysm occlusion.

Chen et al. (14) reported that the use of the Pipeline Flex embolization device for the treatment of intracranial aneurysms is safe and effective, with an occlusion rate of up to 71.3%. Recent studies comparing the Lattice and Pipeline devices have shown that the two devices demonstrate comparable efficacy in the treatment of proximal intracranial aneurysms (86.3% vs. 87.5%) (19). Our findings are consistent with these reports and further extend the evidence to distal intracranial aneurysms.

Flow diverters achieve aneurysm healing by altering intra-aneurysmal hemodynamics, thereby promoting thrombus formation within the aneurysm sac and subsequent endothelialization (20).

Previous studies have suggested that aneurysm healing is influenced by multiple factors, including aneurysm anatomy, aneurysm size, stent deployment characteristics and inflow angle etc. (2, 21–23).

Hemodynamic studies have demonstrated that unfavorable vessel geometry can significantly influence the efficacy of flow diverters by modifying wall shear stress and intra-aneurysmal flow velocity (24). Consistent with these findings, our study suggests that IFAA—defined as the angle between the maximum aneurysm diameter and the centerline of the parent vessel—may serve as an important predictor of aneurysm healing (25, 26). Moreover, the identification of geometric factors such as IFAA may help clinicians better predict treatment outcomes and optimize patient selection.

Traditional flow diverters have a relatively thin distal tip, and the delivery wire may increase the risk of intraparenchymal hemorrhage or distal vessel perforation, particularly when using the push–pull technique in small-caliber or fragile distal arteries. In contrast, the Lattice flow diverter, with its mechanically controlled balloon-assisted system, helps stabilize the distal tip of the delivery wire during deployment, even when the push–pull technique is applied, potentially reducing the risk of arterial perforation (8). In our study, no arterial perforation events were observed in either group during the procedure, although this may be attributable to the limited sample size.

The Lattice device incorporates a proprietary surface modification (MIROR, Metal Interface Reassembly for Optimizing Restenosis), which is designed to reconstruct the metal interface, reduce thrombogenicity, promote endothelialization, and mitigate in-stent restenosis (7). A recent real-world propensity score–matched study demonstrated that the mechanically balloon-assisted LFD with MIROR surface modification was associated with a lower rate of in-stent stenosis (1.8% vs. 14.5%, *p* = 0.037) while achieving perioperative safety, short-term aneurysm occlusion, and clinical outcomes comparable to those of the PED (87.3% vs. 85.5%, *p* > 0.999) (27). The findings of this study suggest that both the Lattice and Pipeline devices can be safely applied in the treatment of distal intracranial aneurysms with satisfactory occlusion rates. These results provide important real-world evidence supporting the effectiveness of the Lattice device.

### Limitations

Several limitations should be acknowledged in this study. First, this was a single-center retrospective study with a relatively small sample size, which may limit the generalizability of the findings. Second, although IPTW was applied to reduce baseline imbalance, residual confounding cannot be completely excluded. Third, the follow-up duration was relatively short, which may have limited the complete assessment of aneurysm occlusion progression, and longer-term outcomes remain to be determined. Future multicenter studies with larger sample sizes and longer follow-up periods are warranted to further validate these findings.

## Conclusion

IPTW analysis and stratified analyses demonstrated comparable efficacy and safety between the Lattice and Pipeline devices in the treatment of distal cerebral aneurysms. Aneurysm geometric and anatomical factors, including the inflow angle (IFAA), aneurysm neck diameter, and branch vessel origin, were independently associated with incomplete or delayed aneurysm occlusion.

## Data Availability

The datasets presented in this study can be found in online repositories. The names of the repository/repositories and accession number(s) can be found in the article/supplementary material.
