# Correction: Comparison of lattice and pipeline flow diverters for the treatment of distal intracranial aneurysms: an inverse probability of treatment weighting analysis

**DOI:** 10.3389/fneur.2026.1941374

**Published:** 2026-07-31

**Authors:** Xinke Liu, Kaiyu Liu, Aoming Jin, Yan Zhao, Tian-Jie Lyu, Wenqi Hu, Yangyang Zhou, Qichen Peng, Xuanping Xie, Xiaoxi Zhu, Siyu Zhou, Jieran Huang, Xinjian Yang, Shiqing Mu

**Affiliations:** 1 Department of Interventional Neuroradiology, Beijing Neurosurgical Institute, Beijing Tiantan Hospital, Capital Medical University, Beijing, China; 2 China National Clinical Research Center for Neurological Diseases, Beijing Tiantan Hospital, Capital Medical University, Beijing, China; 3 School of Mechanical and Engineering, University of Science and Technology Beijing, Beijing, China; 4 Beijing Key Laboratory of Drug and Device Research and Development for Cerebrovascular Diseases, Beijing Tiantan Hospital, Capital Medical University, Beijing, China; 5 Department of Mechanical and Aerospace Engineering, The Hong Kong University of Science and Technology, Tai Po Tsai, Hong Kong SAR, China

**Keywords:** aneurysm, endovascular treatment, flow diverter, occlusion rates, the inflow angle of the aneurysm (IFAA)

In the published article, the order of authors in the author list of the published paper was erroneously given as:

[Shiqing Mu^1*^, Xinke Liu^1†^, Kaiyu Liu^1†^, Aoming Jin^2^, Yan Zhao^3^, Tian-Jie Lyu^2, 4^, Wenqi Hu^5^, Yangyang Zhou^1^, Qichen Peng^1^, Xuanping Xie^1^, Xiaoxi Zhu^1^, Siyu Zhou^1^, Jieran Huang^1^ and Xinjian Yang^1^]

The correct author list reads:

Xinke Liu^1†^, Kaiyu Liu^1†^, Aoming Jin^2^, Yan Zhao^3^, Tian-Jie Lyu^2, 4^, Wenqi Hu^5^, Yangyang Zhou^1^, Qichen Peng^1^, Xuanping Xie^1^, Xiaoxi Zhu^1^, Siyu Zhou^1^, Jieran Huang^1^, Xinjian Yang^1^ and Shiqing Mu^1^^*^

The original version of this article has been updated.

